# Evaluation of the Technicon Axon analyser

**DOI:** 10.1155/S1463924690000311

**Published:** 1990

**Authors:** C. Martínez, M. Márquez, M. Cortés, J. Mercé, J. Rodriguez, F. González

**Affiliations:** Servei de Bioquímica Hospital de la Santa Creu Sant Pau Av. Sant Antoni M. Claret, 167 Barcelona 08025 Spain

## Abstract

An evaluation of the Technicon Axon analyser was carried out following the guidelines of the ‘Sociedad Española de Química Clínica’ and the European Committee for Clinical Laboratory Standards.

A photometric study revealed acceptable results at both 340 nm and 404 nm. Inaccuracy and imprecision were lower at 404 nm than at 340 nm, although poor dispersion was found at both wavelengths, even at low absorbances. Drift was negligible, the imprecision of the sample pipette delivery system was greater for small sample volumes, the reagent pipette delivery system imprecision was acceptable and the sample diluting system study showed good precision and accuracy.

Twelve analytes were studied for evaluation of the analyser under routine working conditions. Satisfactory results were obtained for within-run imprecision, while coefficients of variation for betweenrun imprecision were much greater than expected. Neither specimenrelated nor specimen-independent contamination was found in the carry-over study. For all analytes assayed, when comparing patient sample results with those obtained in a Hitachi 737 analyser, acceptable relative inaccuracy was observed.


Journal of Automatic Chemistry, Vol. 12, No. 6 (November–December 1990), pp. 251–257

### Evaluation of the Technicon Axon analyser

**C. Martínez, M. Márquez, M. Cortés, J. Mercé, J. Rodriguez and F. González**

*Servei de Bioquímica, Hospital de la Santa Creu i Sant Pau, Av. Sant Antoni M. Claret, 167, 08025 Barcelona, Spain*

*An evaluation of the Technicon Axon analyser was carried out following the guidelines of the 'Sociedad Española de Química Clínica' and the European Committee for Clinical Laboratory Standards.*

*A photometric study revealed acceptable results at both 340 nm and 404 nm. Inaccuracy and imprecision were lower at 404 nm than at 340 nm, although poor dispersion was found at both wavelengths, even at low absorbances. Drift was negligible, the imprecision of the sample pipette delivery system was greater for small sample volumes, the reagent pipette delivery system imprecision was acceptable and the sample diluting system study showed good precision and accuracy.*

*Twelve analytes were studied for evaluation of the analyser under routine working conditions. Satisfactory results were obtained for within-run imprecision, while coefficients of variation for between-run imprecision were much greater than expected. Neither specimen-related nor specimen-independent contamination was found in the carry-over study. For all analytes assayed, when comparing patient sample results with those obtained in a Hitachi 737 analyser, acceptable relative inaccuracy was observed.*

### Introduction

The Technicon Axon TM system (Technicon Instruments Corporation, Tarrytown, New York 10591–5097, USA) is a random access biochemistry analyser, which can perform both spectrophotometric and ISE tests. The instrument was evaluated according to the protocol of the 'Sociedad Española de Química Clínica' (SEQC) [1, 2] and the European Committee for Clinical Laboratory Standards (ECCLS) [3]. The evaluation of the analytical units included studies of inaccuracy, imprecision, linearity and drift of the spectrophotometer; imprecision and inaccuracy of the pipette delivery systems (sample and reagents) and of the dilution system; within-run and between-run analytical imprecision, specimen-related and specimen-independent carry-over and relative inaccuracy compared to the results obtained with a Hitachi 737 analyser.

### Materials and methods

*Instrument*

The Technicon Axon is a dual module analyser consisting of the analyser itself and a data processor (workstation). The following types of analysis can be carried out: zero-order and first-order rate, endpoint and blank-corrected (rate and endpoint) analysis.

The workstation allows easy management of the analyser. Features of the workstation include: demographic entry for 972 patients and samples per day; daily and cumulative Westgard quality control; bidirectional host computer communication; over 2000 reaction curves in memory which can be requested at any time; and the ability to visualize reaction curves in real time.

The analytical system contains the hardware and software necessary to perform each analysis (sample tray and reagent trays, reaction cuvettes, spectrophotometer etc.).

The reagent system consists of two trays (2 × 27 reagents with only six positions for user-defined chemistries) and is provided with two syringes (a 1000 µl syringe for aspirating diluent/rinse water and a 500 µl syringe for aspirating/dispensing reagent). Each reagent system, R1 and R2, has its own probe. The first reagent is delivered into the reaction cuvette 37·5 s after the addition of sample. R2 addition time is fixed at 4 min after the addition of R1 to the reaction cuvette. Each reagent tray is divided in two partitions: 21 compartments are kept at between 6 °C and 10 °C, and six compartments are kept at room temperature. The maximum reagent volume is fixed at 400 µl. Up to 38 tests per sample can be requested (36 colorimetric and two ISE).

The delivery of sample into the reaction cuvette is achieved by a process similar to that of reagent delivery. It also consists of two syringes (a 50 µl syringe for aspirating sample and a 500 µl syringe for aspirating sample diluent/rinse water and for dispensing sample and diluent through the probe). Within the sample probe, air is used to separate the sample and rinse water, and also to prevent splashing at the tip when dispensing sample. Sample volumes range from 3 to 47 µl. Up to nine aspirations can be achieved per cup. Up to five automatic dilutions of the sample can be performed. The dilution system allows the operator to use the same chemistries for blood and urine analysis.

The reaction tray contains 90 re-usable, washable, 6 mm light-path Pyrex glass cuvettes. The wash station contains 11 positions, which alternately wash and rinse cuvettes before drying. The sample and reagent probes have their own wash stations where the rinse syringe dispenses a fixed amount of degassed water through the respective probe.

*Table 1. Photometric inaccuracy N = 30, NADH (340 nm).*

| Theoretical O.D. | Observed O.D. | Inaccuracy (%) |
|---|---|---|
| 1·6130 | 1·5335 | −4·93 |
| 1·2130 | 1·1711 | −3·45 |
| 0·8170 | 0·7521 | −7·94 |
| 0·3960 | 0·3831 | −3·26 |
| 0·1360 | 0·1320 | −2·94 |

0142–0453/90 $3.00 © 1990 Taylor & Francis Ltd.

*Table 2. Photometric inaccuracy N = 30, PNP (404 nm).*

| Theoretical O.D. | Observed O.D. | Inaccuracy (%) |
|---|---|---|
| 1·7460 | 1·7504 | 0·25 |
| 1·3660 | 1·3097 | −4·12 |
| 0·9100 | 0·8786 | −3·45 |
| 0·4480 | 0·4376 | −2·32 |

*Table 3. Photometric imprecision N = 30, NADH (340 nm).*

| Mean O.D. | Standard deviation | C.V. (%) |
|---|---|---|
| 1·5335 | 0·0119 | 0·78 |
| 1·1711 | 0·0141 | 1·20 |
| 0·7521 | 0·0171 | 2·27 |
| 0·3831 | 0·0103 | 2·69 |
| 0·1320 | 0·0028 | 2·12 |

The working temperature of the analyser system can be set at either 30 or 37 °C. The temperature is maintained by a hot air bath, which is controlled by heater plates mounted underneath the reaction tray turntable.

The basic analytical process (and thus the maximum incubation time for any chemistry) takes 8 min 45 s. Mixing of reagent and sample takes place two cycles (15 s) after reagent delivery.

Calibration must be accomplished in a special calibration tray (up to 72 positions). Enzyme determinations allow a factor to be employed. A calibrator and water are used for one-point calibration. Moreover, curves can be defined because there are six calibration points with 'n' order polynomial adjustment.

Spectrophotometric analysis is performed by means of a holographic diffraction grating which is located in front of the reaction tray. A tungsten halogen lamp, located in the reagent tray housing, is used to focus light through the reaction cuvette, as it passes by the spectrophotometric read-station. Measurement is possible from a set of 15 wavelengths. For any given chemistry, up to two wavelengths are available for the calculation of a result.

The analytical rate of the system is 480 tests/h for spectrophotometric tests, increasing to 576 tests/h when ISE tests are included.

*Table 4. Photometric imprecision N = 30, PNP (404 nm).*

| Mean O.D. | Standard deviation | C.V. (%) |
|---|---|---|
| 1·7504 | 0·0081 | 0·46 |
| 1·3097 | 0·0074 | 0·57 |
| 0·8786 | 0·0039 | 0·44 |
| 0·4376 | 0·0037 | 0·85 |

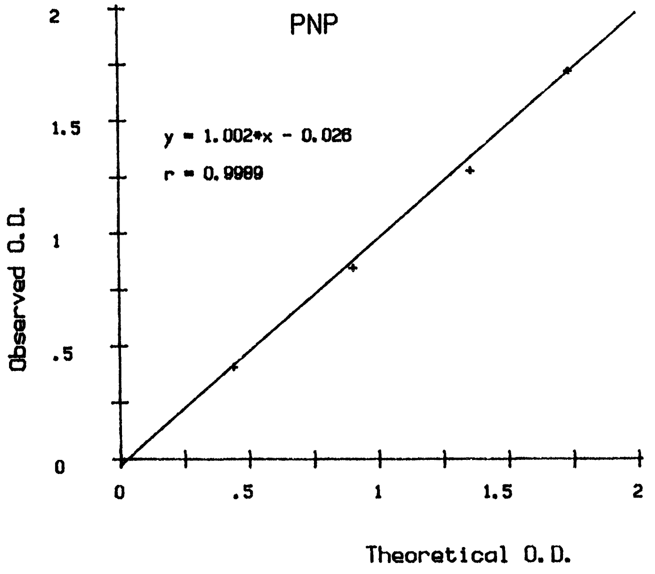

*Figure 1. Photometric linearity, PNP 404 nm.*

*Reagents*

The following reagents were used.

(1) 4-Nitrophenol (PNP) (Merck 6798. E. Merck, Darmstadt, FR Germany).

(2) Sodium hydroxide (NaOH) (Merck 6498).

(3) Nicotinamide Adenine Dinucleotide reduced form (NADH) (Boehringer Mannheim GmbH 107735. Boehringer Mannheim, Mannheim, FR Germany).

(4) Tris(hydroxymethyl)-aminomethane (Tris) (Merck 8382).

(5) Working solutions: PNP 10 mmol/l in NaOH 20 mmol/l; PNP 1 mmol/l in NaOH 20 mmol/l; NADH 3 mmol/l in Tris buffer 80 mmol/l.

The following pre-packaged reagents in individual cassettes available exclusively from Technicon were also used: ALP (AMP buffer, T01-1814-A4); ALT (Tris

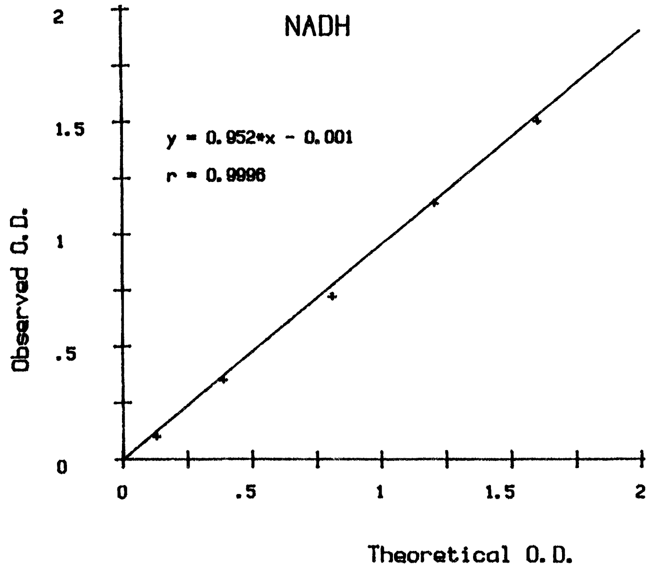

*Figure 2. Photometric linearity, NADH 340 nm.*

*Table 5. Photometric drift.*

| Time | Mean | S.D. | % C.V. |
|---|---|---|---|
| 30 min | 1·3039 | 0·0036 | 0·28 |
| 12 h | 1·3049 | 0·0031 | 0·24 |

buffer IFCC, T01-1760-A4/T11-1762-A5); AST (Tris buffer IFCC, T01-1750-A4/T11-1752-A5); GGT (g-glutamyl-*p*-nitroanilide, T01-1916-A4); LDH (L → P, T01-1967-A8). BUN (urease/GLDH, T01-1823-A4); cholesterol (CHOD/POD, T01-1684-A4); inorganic phosphate (phosphomolybdate UV, T01-1303-A4); glucose (HK/UV, T01-1833-A4), total protein (Biuret, T01-1301-A4); triglycerides (GPO/POD, T01-1863-A4); urate (uricase/POD, T11-2577-A8). The Technicon Set Point calibrator T03-1291-A4 was used.

Reagents used in the Hitachi 737 were from Boehringer Mannheim GmbH Diagnostica: ALP (DEA buffer, 791369/791377); ALT (phosphate buffer IFCC, 791628/791636); AST (phosphate buffer IFCC, 791598/791601); GGT (g-glutamyl-carboxy-nitroanilide, 791644/791652); LDH (P → L, 791733/791741); BUN (urease/GLDH, 791695/791709); cholesterol (CHOD/POD, 791440); inorganic phosphate (phosphomolybdate UV, 804576/804584); glucose (HK/UV, 791571/791580); total protein (Biuret, 791555/791563); triglycerides (GPO/POD, 791768); urate (uricase/POD, 908169/909874). As calibrator, the Boehringer Mannheim calibrator for automated systems 759350 was used.

Control materials were:

(*a*) Test Point I from Technicon, T03-1220-62 (TP I).

(*b*) Test Point II from Technicon, T03-1221-62 (TP II).

(*c*) BioSystems I 18602 from BioSystems S.A., Barcelona, Spain (Bio S).

### Evaluated parameters

*Photometric inaccuracy and photometric imprecision*

Several dilutions of PNP and NADH working solutions were prepared manually. Absorbance was measured at 340 nm for NADH and at 404 nm for PNP. Up to 30 measurements were made for each dilution [4,5]. PNP and NADH solutions were dispensed into the reaction cuvettes through the first reagent delivery system.

*Table 6. Imprecision of sample delivery system PNP (404 nm), N = 36, reagent volume = 350 μl*

| Sample volume | Mean O.D. | Standard deviation | C.V. (%) |
|---|---|---|---|
| 4 μl | 0·4578 | 0·0120 | 2·62 |
| 10 μl | 0·4595 | 0·0095 | 2·06 |
| 20 μl | 0·4478 | 0·0072 | 1·60 |
| 40 μl | 0·4402 | 0·0034 | 0·77 |

Inaccuracy was calculated from the experimental values and the theoretical values obtained from the coefficient of molar absorptivities of NADH and PNP. These theoretical values were checked on a Varian DMS 90 spectrophotometer (Varian Techtron Pty Ltd, Australia).

The mean, standard deviation and coefficient of variation were calculated from 30 successive measurements of each of the solutions prepared as above (NADH and PNP), at 340 nm and at 404 nm respectively.

*Photometric linearity*

Six successive measurements were made from several dilutions prepared manually, plotting the observed mean against the theoretical values from the coefficient of molar absorptivities of PNP and NADH.

*Photometric drift*

To study photometric stability, three consecutive measurements of PNP in NaOH solution, of theoretical absorbance 1·366, were made over the first 30 min and after 12 h, at 404 nm.

*Sample pipette delivery system imprecision*

To check the sample pipette delivery system, a constant volume of NaOH 20 mmol/l was dispensed by the reagent one delivery system, while volumes of PNP solution ranging from 4 to 40 μl were dispensed by the sampler. In each case, the concentration of PNP solution used was calculated to give a final absorbance of around 0·500. Thirty measurements were made to calculate mean, standard deviation and coefficient of variation [6].

*Reagent pipette delivery system imprecision*

In order not to interfere with the reagent pipette delivery system, the sample pipette was blocked. NaOH was dispensed as reagent one and PNP as reagent two. Four trials were run, with volumes ranging from 20 to 350 μl for reagent one and from 350 to 20 μl for reagent two [6]. In order to obtain a more accurate measurement of the reagent pipette delivery system imprecision, the spectrophotometric imprecision was subtracted from the global imprecision: spectrophotometric variance was subtracted from global variance. The spectrophotometric imprecision at these levels of O.D. was calculated from 30 measurements of the final solutions dispensed through the reagent one delivery system.

From this corrected variance, corrected standard deviation and coefficient of variation were calculated.

*Sample dilution system inaccuracy and imprecision*

From a 10 mmol/l PNP solution, three working solutions were prepared; one to check the 1/5 dilution, another for the 1/10 and 1/20 dilutions, and a third for the 1/50 and 1/80 dilutions; these were made up in order not obtain extreme values of O.D. Thirty absorbance measurements were made for each dilution. Inaccuracy was calculated from obtained absorbances and theoretical values from

*Table 7. Imprecision of reagent delivery system, PNP (404 nm), N = 36, sample volume = 0 μl.*

| | | Standard deviation | | | |
|---|---|---|---|---|---|
| R1 vol., R2 vol. | Mean O.D. | Global | Spectr. | Corr. | C.V. (%) |
| 20 μl 350 μl | 1·6123 | 0·0126 | 0·0076 | 0·0100 | 0·62 |
| 120 μl 250 μl | 1·1188 | 0·0102 | 0·0042 | 0·0093 | 0·83 |
| 250 μl 120 μl | 0·5178 | 0·0056 | 0·0028 | 0·0048 | 0·93 |
| 350 μl 20 μl | 0·0635 | 0·0020 | 0·0012 | 0·0016 | 2·52 |

the coefficient of molar absorptivity of PNP at 404 nm. Mean standard deviation and coefficient of variation were calculated to establish imprecision.

### *Analytical imprecision*

To establish within-run imprecision, 30 samples of control sera were tested at three different levels, within the same run. To evaluate the between-run imprecision, one sample of each control serum was processed once a day for 30 days.

### *Specimen-related carry-over*

Following the recommendations of the 'Comisión de Instrumentación de la SEQC' [1,2], sample related carry-over was studied using a permutation order, in which three control samples with different concentrations were distributed along the sample chain. Mean and standard deviation were calculated both for non-contaminated samples and for the whole set of samples.

*Table 8. Sample diluting system imprecision, PNP (404 nm), N = 30.*

| Sample dilution | Mean O.D. | Standard deviation | C.V. (%) |
|---|---|---|---|
| 1/5 | 0·8213 | 0·0067 | 0·82 |
| 1/10 | 0·8931 | 0·0132 | 1·48 |
| 1/20 | 0·4615 | 0·0115 | 2·49 |
| 1/50 | 0·3689 | 0·0079 | 2·14 |
| 1/80 | 0·2312 | 0·0038 | 1·64 |

*Table 9. Sample diluting system inaccuracy, PNP (404 nm), N = 30.*

| Sample dilution | Theoretical O.D. | Observed O.D. | Inaccuracy |
|---|---|---|---|
| 1/5 | 0·8220 | 0·8213 | −0·09 |
| 1/10 | 0·9132 | 0·8931 | −2·20 |
| 1/20 | 0·4566 | 0·4615 | 1·07 |
| 1/50 | 0·3650 | 0·3689 | 1·07 |
| 1/80 | 0·2281 | 0·2312 | 1·36 |

The F value was calculated dividing the variances of all the samples by the variances of the non-contaminated samples.

### *Specimen-independent carry-over*

Following the ECCLS guidelines for the Evaluation of Analyzers in Clinical Chemistry [3], all possible combinations of method sequences for reagents loaded in the same reagent tray were made (reagent one tray: ALT,

*Table 10. Within-run imprecision, N = 30.*

| Analyte | Serum | Mean | S.D. | C.V. (%) |
|---|---|---|---|---|
| ALP U/l 37 °C | TP I | 76 | 2·80 | 3·68 |
| | TP II | 194 | 2·25 | 1·16 |
| | BioS | 223 | 2·14 | 0·96 |
| ALT U/l 37 °C | TP I | 32 | 1·85 | 5·78 |
| | TP II | 118 | 2·14 | 1·81 |
| | BioS | 38 | 1·16 | 3·05 |
| AST U/l 37 °C | TP I | 32 | 1·08 | 3·38 |
| | TP II | 171 | 2·25 | 1·31 |
| | BioS | 23 | 0·99 | 4·30 |
| GGTP U/l 37 °C | TP I | 37 | 0·89 | 2·40 |
| | TP II | 130 | 1·33 | 1·02 |
| | BioS | 77 | 0·91 | 1·18 |
| LDH U/l 37 °C | TP I | 152 | 3·00 | 1·98 |
| | TP II | 458 | 6·38 | 1·39 |
| | BioS | 92 | 1·37 | 1·48 |
| BUN mmol/l | TP I | 7·5 | 0·20 | 2·67 |
| | TP II | 32·5 | 0·70 | 2·15 |
| | BioS | 12·5 | 0·30 | 2·40 |
| Cholesterol mmol/l | TP I | 3·66 | 0·07 | 1·91 |
| | TP II | 5·73 | 0·12 | 2·09 |
| | BioS | 3·69 | 0·05 | 1·36 |
| Inorganic phosphate mmol/l | TP I | 1·01 | 0·02 | 1·98 |
| | TP II | 2·41 | 0·04 | 1·66 |
| | BioS | 1·44 | 0·02 | 1·39 |
| Glucose mmol/l | TP I | 4·1 | 0·04 | 0·98 |
| | TP II | 16·4 | 0·20 | 1·22 |
| | BioS | 9·0 | 0·07 | 0·78 |
| Total protein g/l | TP I | 40·3 | 0·60 | 1·49 |
| | TP II | 70·6 | 1·13 | 1·60 |
| | BioS | 61·9 | 0·26 | 0·42 |
| Triglycerides mmol/l | TP I | 1·77 | 0·04 | 2·26 |
| | TP II | 3·15 | 0·07 | 2·22 |
| | BioS | 1·17 | 0·03 | 2·56 |
| Urate μmol/l | TP I | 308 | 4.07 | 1·32 |
| | TP II | 583 | 8.95 | 1·54 |
| | BioS | 391 | 4·07 | 1·04 |

TP I: Test point I.
TP II: Test point II.
BioS: BioSystems I.

*Table 11. Between-run imprecision, N = 30.*

| Analyte | Serum | Mean | S.D. | C.V. (%) |
|---|---|---|---|---|
| ALP U/l 37 °C | TP I | 78 | 4·54 | 5·82 |
| | TP II | 187 | 7·11 | 3·80 |
| | BioS | 213 | 9·81 | 4·61 |
| ALT U/l 37 °C | TP I | 31 | 1·89 | 6·10 |
| | TP II | 115 | 5·10 | 4·43 |
| | BioS | 38 | 2·52 | 6·63 |
| AST U/l 37 °C | TP I | 31 | 1·93 | 6·23 |
| | TP II | 180 | 5·43 | 3·02 |
| | BioS | 24 | 1·89 | 7·88 |
| GGTP U/l 37 °C | TP I | 38 | 2·17 | 5·71 |
| | TP II | 127 | 3·67 | 2·89 |
| | BioS | 77 | 2·81 | 3·65 |
| LDH U/l 37 °C | TP I | 147 | 7·32 | 4·98 |
| | TP II | 430 | 15·45 | 3·59 |
| | BioS | 86 | 5·57 | 6·48 |
| BUN mmol/l | TP I | 6·9 | 0·41 | 5·94 |
| | TP II | 33·8 | 1·61 | 4·76 |
| | BioS | 11·1 | 0·53 | 4·77 |
| Cholesterol mmol/l | TP I | 3·64 | 0·19 | 5·22 |
| | TP II | 5·61 | 0·23 | 4·10 |
| | BioS | 3·60 | 0·20 | 5·56 |
| Inorganic phosphate mmol/l | TP I | 1·00 | 0·06 | 6·00 |
| | TP II | 2·32 | 0·08 | 3·45 |
| | BioS | 1·38 | 0·06 | 4·35 |
| Glucose mmol/l | TP I | 4·1 | 0·20 | 4·88 |
| | TP II | 15·7 | 0·73 | 4·66 |
| | BioS | 8·8 | 0·42 | 4·77 |
| Total protein g/l | TP I | 36·1 | 2·37 | 6·56 |
| | TP II | 63·9 | 3·06 | 4·79 |
| | BioS | 56·2 | 2·73 | 4·86 |
| Triglycerides mmol/l | TP I | 1·71 | 0·10 | 5·85 |
| | TP II | 2·87 | 0·13 | 4·53 |
| | BioS | 1·05 | 0·07 | 6·67 |
| Urate μmol/l | TP I | 313 | 9·52 | 3·04 |
| | TP II | 587 | 20·42 | 3·48 |
| | BioS | 386 | 20·06 | 5·20 |

TP I: Test point I.
TP II: Test point II.
BioS: BioSystemsI.

AST, glucose, cholesterol, urate, inorganic phosphate and total protein) (reagent two tray: ALT, AST, BUN, triglycerides, ALP, LDH and GGT).

*Relative inaccuracy*

Passing Bablok regression analysis was used to compare results of patient samples obtained in the Axon system with those obtained in the Hitachi 737 [7,8].

### Results and discussion

*Photometric inaccuracy*

Results of photometric inaccuracy at 340 and at 404 nm are shown in tables 1 and 2 (respectively); the tables show the values of theoretical and observed absorbances and percentage inaccuracy. The bias observed at 404 nm ranged from −4·12% to 0·25%, and at 340 nm from −7·94% to −2·94%. Both inaccuracies were acceptable.

*Photometric imprecision*

The results of photometric imprecision are shown in tables 3 and 4. Coefficients of variation obtained at 404 nm were always below 1% and those observed at 340 nm ranged from 0·78% to 2·12%, being greater at low absorbances.

*Photometric linearity*

Results are shown in figures 1 and 2. Linearities for NADH at 340 nm, and for PNP at 404 nm, were acceptable.

*Photometric drift*

The results of photometric drift evaluation are presented in table 5. The PNP solution read at 30 min and 12 h at 404 nm showed similar mean absorbances and standard deviations. Thus, drift can be considered to be negligible.

*Sample pipette delivery system imprecision*

As shown in table 6, the imprecision was acceptable in the range of sample volumes studied, although it increased when the dispensed sample volume diminished.

*Reagent pipette delivery system imprecison*

The results presented in table 7 show the mean, the global, spectrophotometric and corrected standard deviation, and the coefficient of variation which ranged within 0·62% and 2·54%. As with photometric imprecision, the lower the absorbances, the greater the imprecision.

*Sample diluting system inaccuracy and imprecision*

Tables 8 and 9 show the results of imprecision and inaccuracy respectively. Imprecision ranges between 0·82% and 2·49%, and inaccuracy between −2·20% and 1·36%. Both were acceptable in all cases.

*Analytical imprecision*

Tables 10 and 11 show the results of within and between-run analytical imprecision respectively. For all studied analytes, the within-run imprecision was acceptable. This contrasts with some values obtained for between-run imprecision which were surprisingly high.

*Specimen-related carry-over*

The results of carry-over studies are presented in table 12, which shows the mean and standard deviation for all samples (mean 1, SD 1), and for non-contaminated samples (mean 2, SD 2), for each analyte and level. The calculated F value is also expressed. In all cases, the calculated F value was less than the value of an F distribution for a $p = 0{\cdot}05$. This value for high and low levels (16 non-contaminated samples of 24 samples) is 2·30, and for medium level (nine non-contaminated samples of 12 samples) it is 3·31. Thus we can conclude that there is no significative sample-related carry-over.

*Table 12. Specimen-related carry-over.*

| Analyte | Level | Mean 1 | S.D. 1 | Mean 2 | S.D. 2 | F. Calc. |
|---|---|---|---|---|---|---|
| ALP | High | 680 | 20·78 | 680 | 20·47 | 1·03 |
| | Medium | 432 | 15·30 | 432 | 15·30 | 1·00 |
| | Low | 237 | 9·02 | 236 | 9·49 | 0·90 |
| ALT | High | 137 | 4·26 | 137 | 4·68 | 0·83 |
| | Medium | 86 | 2·90 | 86 | 3·05 | 0·91 |
| | Low | 43 | 2·37 | 43 | 2·70 | 0·77 |
| AST | High | 147 | 6·25 | 149 | 6·40 | 0·95 |
| | Medium | 95 | 3·75 | 95 | 3·41 | 1·21 |
| | Low | 45 | 3·19 | 44 | 3·53 | 0·82 |
| GGTP | High | 194 | 10·67 | 193 | 11·65 | 0·84 |
| | Medium | 125 | 10·03 | 125 | 10·37 | 0·94 |
| | Low | 66 | 3·62 | 66 | 3·79 | 0·91 |
| LDH | High | 395 | 29·08 | 393 | 32·67 | 0·79 |
| | Medium | 259 | 15·97 | 260 | 17·31 | 0·85 |
| | Low | 139 | 7·63 | 138 | 7·49 | 1·04 |
| BUN | High | 27·9 | 0·97 | 27·9 | 0·75 | 1·67 |
| | Medium | 17·7 | 0·52 | 17·7 | 0·59 | 0·78 |
| | Low | 9·1 | 0·40 | 9·1 | 0·43 | 0·87 |
| Chol. | High | 6·65 | 0·15 | 6·64 | 0·15 | 1·00 |
| | Medium | 4·27 | 0·09 | 4·29 | 0·08 | 1·27 |
| | Low | 2·21 | 0·06 | 2·22 | 0·06 | 1·00 |
| In. phos. | High | 2·87 | 0·03 | 2·87 | 0·04 | 0·56 |
| | Medium | 1·83 | 0·02 | 1·82 | 0·02 | 1·00 |
| | Low | 0·97 | 0·09 | 0·98 | 0·10 | 0·81 |
| Glucose | High | 19·5 | 0·22 | 19·5 | 0·22 | 1·00 |
| | Medium | 12·4 | 0·23 | 12·4 | 0·25 | 0·85 |
| | Low | 6·5 | 0·09 | 6·5 | 0·10 | 0·81 |
| T. protein | High | 121·2 | 1·14 | 121·0 | 1·03 | 1·22 |
| | Medium | 77·3 | 1·22 | 77·3 | 1·41 | 0·75 |
| | Low | 39·8 | 0·99 | 39·7 | 0·87 | 1·29 |
| Triglyc. | High | 3·21 | 0·05 | 3·22 | 0·05 | 1·00 |
| | Medium | 2·10 | 0·03 | 2·10 | 0·03 | 1·00 |
| | Low | 1·14 | 0·03 | 1·14 | 0·03 | 1·00 |
| Urate | High | 904 | 10·99 | 905 | 12·15 | 0·82 |
| | Medium | 609 | 10·32 | 606 | 8·26 | 1·56 |
| | Low | 354 | 9·22 | 351 | 7·88 | 1·37 |

*Specimen-independent carry-over*

In all method sequence combinations, the carry-over effect measured was less than twice the within-run imprecision of the method studied and can therefore be ignored.

*Relative inaccuracy*

Table 13 shows the number of pairs compared, the studied range, slope and *y* intercept with their confidence intervals, and the regression coefficient (*r*) for each analyte.

The marked deviations of the slope from 1 for ALP and LDH are due to methodological differences.

### Conclusion

In general terms, the evaluation of the instrument was satisfactory, and, in our opinion, it fulfills the requirements for use in routine-work in a medium-size laboratory.

The photometer showed a slight tendency to give low absorbance values at 340 nm.

The photometric imprecision was acceptable at the two wavelengths studied and no photometric drift was found over a 12 h working period.

The reagents and sample delivery system showed a correct imprecision and the dilution system showed an acceptable inaccuracy and imprecision.

The analytical imprecision study showed an acceptable within-run imprecision, which was especially low for non-enzymatic analytes, while the between-run imprecision was a little bit higher than expected for those analytes. This might not be due to the instrument, but, rather, to the quality of the reagents.

The real analytical rate of the system was similar to the theoretical rate specified by the manufacturer.

### References

1. Galimany, R., Alsina, M. J., Biosca, C., Calvet, M., Castiñeiras, M. J., Esquerdo, E., Fuster, M., Lema, F., Martínez, M., Mateo, J., Navarro, J. M. and Paz, M., *Boletín Informativo S.E.Q.C.*, **34** (1986), 3.

*Table 13. Relative inaccuracy.*

| Analyte (Range) | No. of pairs | Slope (Confidence intervals) | *Y* intercept (Confidence intervals) | *r* |
|---|---|---|---|---|
| ALP | 127 | 0·364 | −3·000 | 0·995 |
| (69–2734) | | (0·357/0·374) | (−4·538/−1·481) | |
| ALT | 115 | 1·043 | 3·261 | 0·999 |
| (7–1353) | | (1·028/1·059) | (2·529/3·889) | |
| AST | 125 | 0·868 | 0·709 | 0·993 |
| (11–948) | | (0·844/0·900) | (0·000/1·344) | |
| GGT | 126 | 1·030 | −0·500 | 0·999 |
| (9–868) | | (1·002/1·047) | (−1·039/0·910) | |
| LDH | 126 | 0·447 | 13·303 | 0·962 |
| (169–6740) | | (0·434/0·462) | (8·102/17·56) | |
| BUN | 126 | 1·000 | 0·300 | 0·989 |
| (2·1–33·0) | | (0·976/1·040) | (0·032/0·418) | |
| Cholest. | 127 | 1·036 | −0·036 | 0·993 |
| (1·25–9·69) | | (1·014/1·062) | (−0·147/0·052) | |
| In. phosp. | 125 | 1·138 | −0·213 | 0·906 |
| (0·60–2·38) | | (1·050/1·231) | (−0·328/−0·100) | |
| Glucose | 124 | 1·000 | −0·100 | 0·992 |
| (3·5–15·9) | | (0·958/1·000) | (−0·100/0·133) | |
| Tot. prot. | 81 | 1·034 | −3·931 | 0·978 |
| (45·8–97·3) | | (0·982/1·089) | (−7·549/−0·374) | |
| Triglyc. | 108 | 1·195 | 0·036 | 0·995 |
| (0·36–8·26) | | (1·147/1·250) | (−0·020/0·095) | |
| Urate | 127 | 1·056 | 5·778 | 0·960 |
| (47–686) | | (1·000/1·122) | (−16·54/28·00) | |

2. Galimany, R., Alonso, J. R., Barrio, J. M., Castiñeiras, M. J., Esquerdo, E., Fernandez, E., Garcia-Merlo, S., Lema, F., Mateo, J., Paz, M. and Roura, F., *Boletín Informativo de la S.E.Q.C.* (1980).
3. Haeckel, R., Busch, E. W., Jennings, R. D., Kokholm, G., Truchaud, A., *Guidelines for the Evaluation of Analysers in Clinical Chemistry* (ECCLS Document), Vol. 3, 2 (1986).
4. Grafmeyer, D. and Dingeon, B., *Information Scientifique du biologiste*, **6** (1984), 376–378.
5. Vanderlinde, R. R., Richards, A. H. and Kowalski, P., *Clinica Chimica Acta*, **61** (1975), 39.
6. Geary, T. D., *Clinical Biochemistry Reviews*, **2** (1981), 64.
7. Passing, H. and Bablok, W., *Journal of Clinical Chemistry and Clinical Biochemistry*, **21** (1983), 709.
8. Bablok, W. and Passing, H., *Journal of Automatic Chemistry*, **7** (1985), 74.

